# Hospital Expenditure.—The Commissariat

**Published:** 1896-06-06

**Authors:** 


### THE INSTITUTIONAL WORKSHOP.

### HOSPITAL EXPENDITURE.—THE COMMISSARIAT.

### XVI.—THE PRICE OF MEAT.

NOTHING is more remarkable in the management of large London institutions than the variation in prices paid for articles of common consumption. In London all hospitals have equal advantages with regard to their food supplies; it may be safely asserted that in none, at the present time, is the quality of the food distinctly bad, while few secretaries or managers would be willing to admit the existence of too high a standard of quality; and yet the complete summary of prices paid in 1893, courteously communicated from nearly all the leading hospitals, reveals a difference amounting in many instances to more than 100 per cent. in the cost of the commonest articles. The variations in the cost of meat are, perhaps, the most striking, and certainly the most important. In London the contract system reigns supreme, but it is not uniformly worked on the same principle. The majority of institutions require a separate specification for every part of the animal likely to be required, and this runs sometimes in all to as many as twenty items. A few contract for the main supplies at one price, and require a separate specification for "best joints for officers' tables." Four hospitals level the contract down to two prices, and in two only is one price quoted for the whole supply.

At the very outset of this inquiry it becomes apparent that the key to much of the variation in prices lies at the root of the vexed question of English versus foreign meat. Needless to say, that as this is a question depending far more on opinion than on ascertained fact, the hospitals reflect very adequately the various shades of popular dogma on the subject, the only point of agreement being the absolute certainty with which the individual theory is maintained. The following verdicts relating to the use of foreign meat in hospitals have been elicited from various secretaries and managers of wide hospital experience.

A uses nothing but English meat; believes foreign meat to be almost valueless, and states that it is a well-known fact that families using foreign meat exclusively are attacked by anæmia and slowly lose their vital powers.

B uses nothing but English meat; pays a higher price, but believes it to be the truest economy.

C prefers English meat, and professes to use nothing else; contracts for it, however, at a very low price, and has more than a suspicion that foreign meat is often delivered; does not see how this can be stopped.

D uses English meat for the officers' table, foreign for nurses, household, and patients; a variety of this custom includes nurses among those provided with English meat.

E uses English meat only for beef tea; states it to be notorious that foreign meat is no good for beef tea; for all other purposes uses foreign meat, which is excellent.

F uses foreign meat, which gives satisfaction to everyone, exclusively; conceals the fact, however, owing to the existence of a strong prejudice against foreign meat in the hospital.

G openly uses foreign meat only; understands the different qualities, stipulates for the best, and finds it perfectly satisfactory.

H uses the cheapest meat to be had; is satisfied all foreign is as good as English, and knows nothing of varieties.

We shall have something to say on this question later on. At present we are concerned merely with the extent to which this controversy affects prices. If all hospitals understood something of the meat trade prices would fall under distinct headings, English or foreign, and the matter would be simple enough. But the question is heavily complicated by ignorance and prejudice, with the result that every possible gradation of price from 2d. up to 8d. and 10½d. is paid per lb., with small distinction of quality or description. A brief examination of some of the contract prices will illustrate this point.

The two hospitals which contract under one price for their meat supply pay almost precisely the same viz., 5¾d. and 5⅞d. per lb. all round. This is, of course foreign meat price, and is a very fair all-round quotation for a supply of the best foreign meat of all descriptions.

Of those who contract under two prices, No. 3 is again obviously foreign. The two prices are 5d. and 5½d., and the slightly higher price is paid for the nurses' meat. The difference is too small, it may be surmised, to produce any appreciable rise in quality, and it may be remarked that if an inferior quality of foreign meat is supplied for the servants and patients 5½d. per lb. all round is too high a price to give.

No. 4 pays 4½d. for shin, &c., and 6½d. for joints stipulating for English meat. The fallacy of this stipulation is easy to be seen. No contractor could afford to supply good English meat at a price so far below the ordinary market value; good English shin of beef, even in large quantities, can hardly be supplied below 7d. per lb., and English joints of satisfactory quality will not be less than 8d. The price is, in fact, too low for English meat and too high for foreign, since excellent American shin can be had for 4d. per lb., and the best foreign joints, as we have seen above, for under 6d.

No. 5 pays 4½d. for foreign meat all round and 7d. for English joints for the officers' table. It is certain that foreign meat of the best quality could not be supplied at the former price. It may easily, however, although inferior in quality, be perfectly sound and fit for consumption. But the custom of supplying the patients with meat inferior in quality to that of other members of the household is unsound in principle and, we believe, expensive in practice. It cannot be contended that the patients are indifferent about quality, for none are more critical in their purchase of food than the poor. While again, if the meat supplied for their use is in all respects satisfactory, as it ought to be, why pay a higher price for that supplied to nurses and officers?

No. 6 pays 6d. and 8½d. per lb., evidently for English meat. For these prices, but hardly below them, the contractor may reasonably be expected to supply good meat from none but home-killed animals. Probably ewe-mutton and cow-beef will sometimes be supplied, but if the meat is well hung the consumer is not likely to notice much difference. It may be safely asserted that no contract for English meat at one price could be honestly carried out at less than 8d. per lb. all round.

It is impossible to notice in detail the prices paid at those institutions where each joint is contracted for separately. It will, however, be interesting to observe the prices paid per lb. in various London hospitals for the stock hospital necessaries, leg of mutton and shin of beef.

| Shin of Beef. | Leg of Mutton. |
|---|---|
| 3¼d. | 3½d. |
| 3½d. | 4⅛d. (all-round price). |
| 4⅛d. (all-round price). | 4½d. |
| 4½d. | 5d. |
| 5d. | 5¼d. |
| 5¼d. | 5½d. |
| 5¾d. (all-round price). | 5¾d. (all-round price). |
| 5⅞d. " " | 5⅞d. " " |
| 6½d. | 6½d. |
| | 7d. (all-round price). |
| | 7½d. |
| | 8d. |
| | 8½d. |

Seeing that the difference as regards the mutton amounts to as much as 5d. per lb. between the two extremes the importance of this matter becomes at once apparent. In a hospital containing from 200 to 300 beds at least 15,000 lbs. of leg of mutton will be consumed in a year, and the difference between the two prices works out for this one joint alone at a total of £300 per annum. Of shin of beef in a hospital of this size not less than 25,000 lbs. will be required, and the difference of 2⅜d. between the extremes again amounts to very nearly as much, viz., close upon £300.

